# Kinetic mRNA Profiling in a Rat Model of Left-Ventricular Hypertrophy Reveals Early Expression of Chemokines and Their Receptors

**DOI:** 10.1371/journal.pone.0161273

**Published:** 2016-08-15

**Authors:** Simona Nemska, Laurent Monassier, Max Gassmann, Nelly Frossard, Reza Tavakoli

**Affiliations:** 1 Institute of Veterinary Physiology and Zurich Center of Integrative Human Physiology, University of Zurich, Zurich, Switzerland; 2 Laboratoire d’Innovation Thérapeutique, UMR7200, Université de Strasbourg—CNRS, Strasbourg, France; 3 Laboratoire de Neurobiologie et Pharmacologie Cardiovasculaire EA7296, Fédération de Médecine Translationnelle, Université de Strasbourg, Strasbourg, France; 4 Department of Cardiac Surgery, Canton Hospital Lucerne, Lucerne, Switzerland; "INSERM", FRANCE

## Abstract

Left-ventricular hypertrophy (LVH), a risk factor for heart failure and death, is characterized by cardiomyocyte hypertrophy, interstitial cell proliferation, and leukocyte infiltration. Chemokines interacting with G protein-coupled chemokine receptors may play a role in LVH development by promoting recruitment of activated leukocytes or modulating left-ventricular remodeling. Using a pressure overload-induced kinetic model of LVH in rats, we examined during 14 days the expression over time of chemokine and chemokine receptor mRNAs in left ventricles from aortic-banded *vs* sham-operated animals. Two phases were clearly distinguished: an inflammatory phase (D3-D5) with overexpression of inflammatory genes such as *il-1ß*, *tnfa*, *nlrp3*, and the *rela* subunit of *nf-kb*, and a hypertrophic phase (D7-D14) where *anp* overexpression was accompanied by a heart weight/body weight ratio that increased by more than 20% at D14. No cardiac dysfunction was detectable by echocardiography at the latter time point. Of the 36 chemokines and 20 chemokine receptors analyzed by a Taqman Low Density Array panel, we identified at D3 (the early inflammatory phase) overexpression of mRNAs for the monocyte chemotactic proteins CCL2 (12-fold increase), CCL7 (7-fold increase), and CCL12 (3-fold increase), for the macrophage inflammatory proteins CCL3 (4-fold increase), CCL4 (2-fold increase), and CCL9 (2-fold increase), for their receptors CCR2 (4-fold increase), CCR1 (3-fold increase), and CCR5 (3-fold increase), and for CXCL1 (8-fold increase) and CXCL16 (2-fold increase). During the hypertrophic phase mRNA expression of chemokines and receptors returned to the baseline levels observed at D0. Hence, this first exhaustive study of chemokine and chemokine receptor mRNA expression kinetics reports early expression of monocyte/macrophage-related chemokines and their receptors during the development of LVH in rats, followed by regulation of inflammation as LVH progresses.

## Introduction

Left-ventricular hypertrophy (LVH) commonly follows chronic pressure or volume overload in diseases such as essential arterial hypertension and aortic valve stenosis. Insufficient relief of the left-ventricular workload will ultimately result in an irreversible state of cardiac dysfunction leading to arrhythmias, heart failure, or even death. Numerous stress stimuli can increase cardiomyocyte protein synthesis and cell volume [1–3]; these changes are accompanied by energy metabolism deficits, vascular dysfunction, and alterations of the extracellular matrix composition [4–6]. In addition, pressure overload causes interstitial cell proliferation (vascular smooth muscle and endothelial cells and fibroblasts) that causes increased vascular stiffness [7, 8], accompanied by inflammatory signals generated by the vessel wall (endothelium) [9–11]. The role of inflammatory response in cardiac hypertrophy was first suggested by *in vitro* studies of rat neonatal cardiomyocytes, where activation and nuclear translocation of NF-kB (nuclear factor kB) are required for hypertrophic cardiomyocyte growth [12, 13]. Macrophages and neutrophils are reported to accumulate in the hypertrophic left ventricle of a mouse model of aortic constriction 3–6 days after surgery [14, 15]. Similarly, the observation of newly recruited leukocytes in biopsies from hypertrophic hearts of patients with aortic valve stenosis suggests that hypertrophy is associated with a persistent proinflammatory response [16], which in turn has been related to the production of profibrotic factors (TGF-ß, transforming growth factor-ß) by newly infiltrated immune cells [17, 18].

In proinflammatory disease conditions, the trafficking of immune cells to sites where inflammatory stress is occurring is regulated mainly by the action of chemokines binding to their G protein-coupled receptors (GPCR) [19–21]. In cardiac hypertrophy, upregulated expression of a small number of chemokines is reported both in animal models [22, 23] and in patients with cardiac hypertrophy [24–26]. Elevated levels of CCL2 are found in cardiac biopsies from patients with cardiac hypertrophy due to aortic stenosis [16]. Similarly, CXCL16 is found at high levels in the plasma of patients with right-ventricular hypertrophy due to pulmonary stenosis and those with LVH in heart failure [27, 28]; in the latter case, expression levels correlated with disease severity. In addition, CX3CL1, an atypical chemokine that, like CXCL16, exists in both membrane-bound and soluble forms, is also detected in hearts and serum of patients with heart failure [26, 29]. Levels of circulating CCL2, CCL3, and CCL5 chemokines are high in patients with cardiac hypertrophy and congestive heart failure [24], and CCL21 is found at significantly higher than normal levels in the serum of patients with LVH due to aortic stenosis and pressure overload [30]. Finally, high CXCL12 plasma levels are found in patients with hypertrophic cardiomyopathy, LVH, and heart failure, and CXCL12-dependent fibrocyte migration increases when diffuse fibrosis is present [31, 32]. These data suggest that chemokine and chemokine receptor interactions play a pivotal role in the establishment of cardiac hypertrophy.

To obtain deeper insight into the molecular factors associated with LVH development, we undertook a comprehensive analysis of mRNA expression of chemokines and their receptors in the initiation and progression phases of cardiac hypertrophy in a rat model of aortic banding.

## Material and Methods

### Animals

Male Lewis rats weighing 100–110 g were purchased from Janvier (Le Genest-Saint-Isle, France) and maintained on a 12/12-hour light/dark cycle, in standard T3 cages, with food and water available *ad libitum*. In this protocol no analgesia or antibiotics were given as the opioid analgesics and anti-inflammatory drugs may impair the inflammatory response during hypertrophy. Antibiotics can alter the expression of chemokines, which might interfere with our results [33, 34]. Animal experimentation was performed in accordance with the national guidelines, and the protocols were approved by the "Strasbourg Regional Ethics Committee for Animal Experimentation CREMEAS” (#02438.01) in accordance with European regulations.

### Aortic banding

Aortic banding was performed as described [3]. Briefly, rats were anesthetized by pentobarbital (Ceva, 6742145; 50 mg/kg i.p. in saline). After laparotomy, a polyurethane ring 1 mm in diameter was placed around the suprarenal abdominal aorta to create stenosis. Sham operations were performed without ring placement. The abdominal wall was sutured with a 4.0 surgical suture (Ethicon, USA). After operation, animals are placed on a heating plate (35°C) until they wake up. Rats were then kept individually in T3 cages. The survival rate in this study was 88%; fourteen of the animals (all from aortic-banded group) died during the experiment. Five out of 14 animals did not wake up after surgery and died from technical surgical failure on day (D) 0 of surgery. Five rats were found unexpectedly dead in the morning on D4-D6 post-operation. Four rats had to be euthanized by 100 mg/Kg pentobarbital because of unhealthy appearance with red and sunken eyes, ruffled hair, hunched body and/or changes in the locomotion on D4-D6 post-operation, most probably due to hypertensive crisis. Animal monitoring was performed twice a day. Body weight did not differ at any point during the study between these rats and the sham-operated group. Hearts were harvested on D0, 1, 3, 5, 7, and 14 from n = 6 rats in each group; their atria were removed and the ventricles weighed. Right ventricles were removed and a transversal slice of the left ventricle (LV) was cut and paraffin-embedded for histological study. The remaining part of the LV was frozen in liquid nitrogen for biochemical analyses.

### Echocardiography

Serial echocardiographic-Doppler assessment of cardiac anatomy, function, and hemodynamics was performed on D0, 1, 3, 5, 7, and 14 after operation (n = 6 per group). Rats were weighed, anesthetized by pentobarbital (40 mg/kg i.p.), and studied with a Sonos 5500 echocardiography machine (Philips, USA) equipped with a 12-MHz sectorial transducer. The animal's body temperature was maintained at 37°C with a controlled heating pad (homeothermic blanket control unit, Harvard Apparatus). Interventricular septum and posterior wall thickness were measured on the parasternal short-axis view of the LV in diastole. The end-diastolic and end-systolic left ventricle diameters (EDLVD and ESLVD) were measured on the parasternal LV short-axis view from M-mode. The fractional shortening (FS = [(EDLVD-ESLVD) / EDLVD] x100) and the ejection fraction (EF = [(EDLVD^3^-ESLVD^3^)/EDLVD^3^] x 100) were calculated. All measured and calculated indices are means of four consecutive beats.

### Histology

A transversal slice from the basal part of the LV tissue (n = 6 per group) was fixed in paraformaldehyde 4% for 24 h and paraffin-embedded after successive lavages in ethanol (70–100%) for 1 h each and incubation in xylene for 3 h. Then 5-μm sections were cut and mounted on Superfrost glass slides (Fisher Scientific). After deparaffinization in xylene and successive rehydration, sections were stained with hematoxylin-eosin. The LV wall area and the LV cross-sectional thickness (15 measurements for each section) were measured with ImageJ 1.47v software. Sections were observed on a Leica DM4000B microscope with an Olympus DP72 camera and analyzed with CellSensDimension software.

### RNA extraction, reverse transcription, and qPCR

Total RNA was extracted both from the apex and the base of the LV (30–40 mg tissue for each) with TriReagent® (Euromedex) and purified after binding on silica columns under high-salt conditions (RNeasy Mini kit, Qiagen). RNA quantity and purity were assessed by absorption spectra analysis (Nanodrop®) and fluorescence absorption (Qubit® Fluorimetric RNA assay, Invitrogen). RNA quality was evaluated by the absorption ratios A260/A280 and A260/A230 between 1.8 and 2.2. The concentration ratio between Qubit® and Nanodrop® ranged from 0.9 to 1.1. RNA integrity was analyzed with the Agilent 2100 Bioanalyzer with an RNA 6000 Chip and 2100 Expert software (Agilent Technologies). All samples had an RNA integrity number higher than 8. RNA (1 µg) was then reverse-transcribed with the high-capacity cDNA Reverse Transcription kit containing MultiScribe Reverse Transcriptase from the murine moloney leukemia virus (Applied Biosystems). Real-time qPCR was performed on the ABIPrism 7900 Thermocycler (Applied Biosystems) with 384-well Taqman Low Density arrays (Applied Biosystems) designed for the analysis of the 36 chemokines and 20 chemokine receptors for which primers were then available for rats (S1 Table). We also analyzed nine inflammatory genes (*crp* (c-reactive protein), *il-1ß* (interleukin-1ß), *il-6*, *il-17a*, *ifng* (interferon g), tnfa (tumor necrosis factor a), *nlrp3* (NOD-like receptor family, pyrin domain containing 3), *scf* (stem cell factor, kit ligand), and *rela*) and five housekeeping genes: *gapdh* (glyceraldehyde-3-phosphate dehydrogenase), *18S*, *hprt1* (hypoxanthine phosphoribosyltransferase), *hmbs* (hydroxyl-methylbilane synthase), and *tbp* (TATA box binding protein) (S1 Table). The stability of the reference genes was analyzed (DataAssist®, Applied Biosystems), with lower scores indicating more stable reference genes [35]. The lowest score was observed for *gapdh* (0.84). Other samples showing good Ct stability were *18S* (0.89), *hprt1* (0.89), and *hmbs* (0.86) (S1 Fig). The only unsatisfactory score was 2.22 for *tbp*. Results were therefore referenced to the most stable gene, *gapdh*, and compared to those referenced to the geometric mean of the 4 stable genes: both analyses showed similar results. The endogenous regulators of cardiac hypertrophy *anp* (atrial natriuretic peptide) and *bnp* (brain natriuretic peptide) were used as positive controls for the hypertrophic phenotype. The cutoff for gene expression was set at Ct<32. Results are presented as mean mRNA expression in the qPCR results from each part of the LV base and apex.

### Data analysis and statistics

Statistical analyses were performed by one-way ANOVA followed by Bonferroni‘s multiple comparison tests. Differences in the LV area and wall thickness for the two groups were compared by a *t*-test. Means were considered statistically significantly different when *P*<0.05.

## Results

### Inflammation occurs before hypertrophy in the rat LVH model

**Fig 1:**
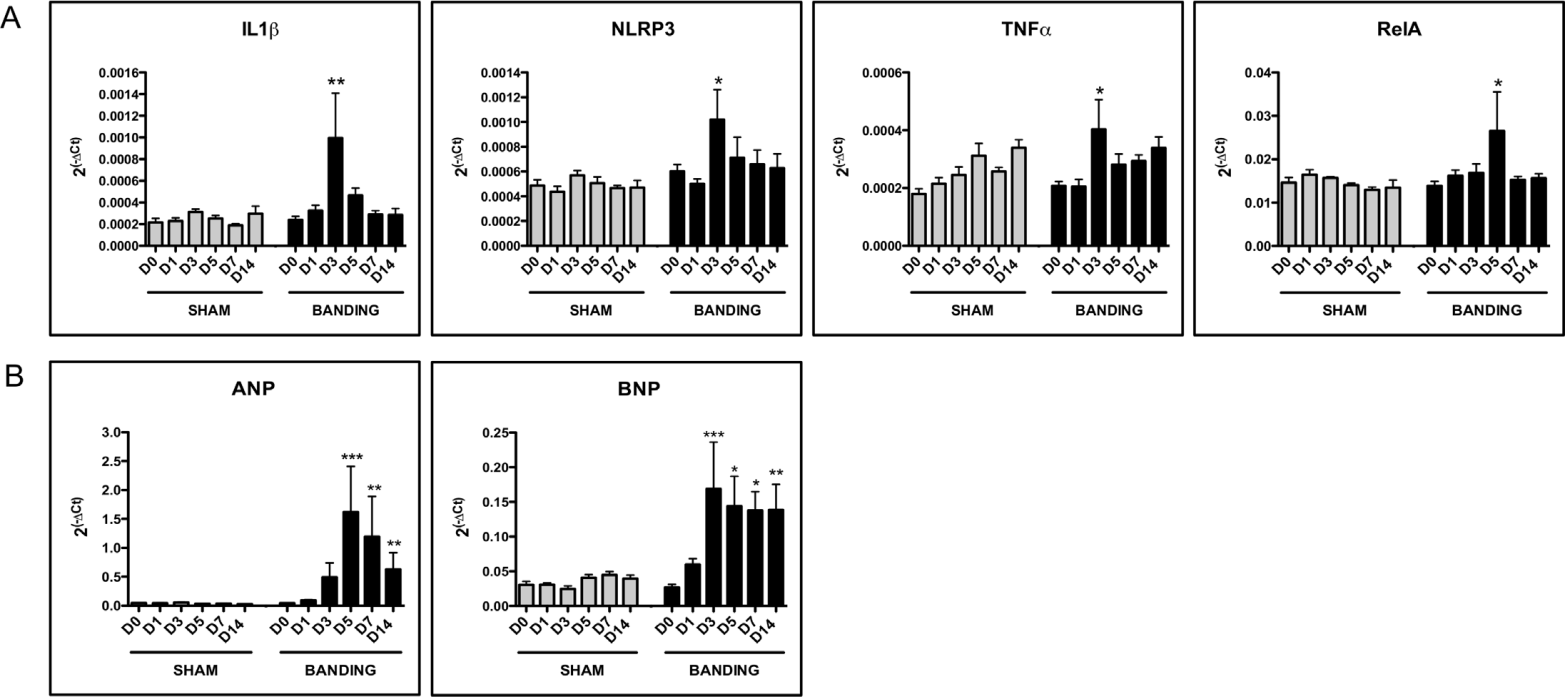
Inflammatory and hypertrophic genes mRNA expression in rat LVH. (A) mRNA overexpression of inflammatory genes in aortic-banded *vs* sham-operated groups (n = 6 per group). (B) mRNA expression of the atrial natriuretic protein (ANP) and of the brain natriuretic peptide (BNP), positive controls for cardiac hypertrophy in aortic-banded *vs* sham-operated animals (n = 6 per group). Expression is calculated as 2^(-ΔCt)^ where the calibrator is the mRNA level of the *gapdh* reference gene. Data are presented as mean ± SEM. *P<0.05 **P<0.01 compared to sham group.

We performed a kinetic study of the mRNA expression of pro-inflammatory and pro-hypertrophic genes in the aortic-banded model. At D0, the pro-inflammatory genes *il-1ß*, *tnfa*, *nlrp3*, *scf*, and *rela* were expressed both in aortic-banded and sham groups (Ct<32), whereas *crp*, *il-6*, *il-17a*, and *ifng* were not (Fig 1A). At D3, expression of IL-1ß and TNFa mRNA increased by 3.4±1.5-fold (*P*<0.01) and 1.9±0.6-fold (*P*<0.05), respectively, in aortic-banded *vs* sham-operated animals (Fig 1A). NLRP3 mRNA that encodes a protein of the inflammasome, also increased at D3 by 1.9±0.5-fold (*P*<0.05) (Fig 1A). At D5, expression of the RelA subunit of NF-kB was 1.7±0.4-fold higher in the aortic-banded rats (*P*<0.05). Expression of SCF did not change over time and between aortic-banded and sham-operated animals (e.g. 0.88±0.14-fold at D3 and 1.08±0.11-fold at D5) (S2 Fig).

**Fig 2:**
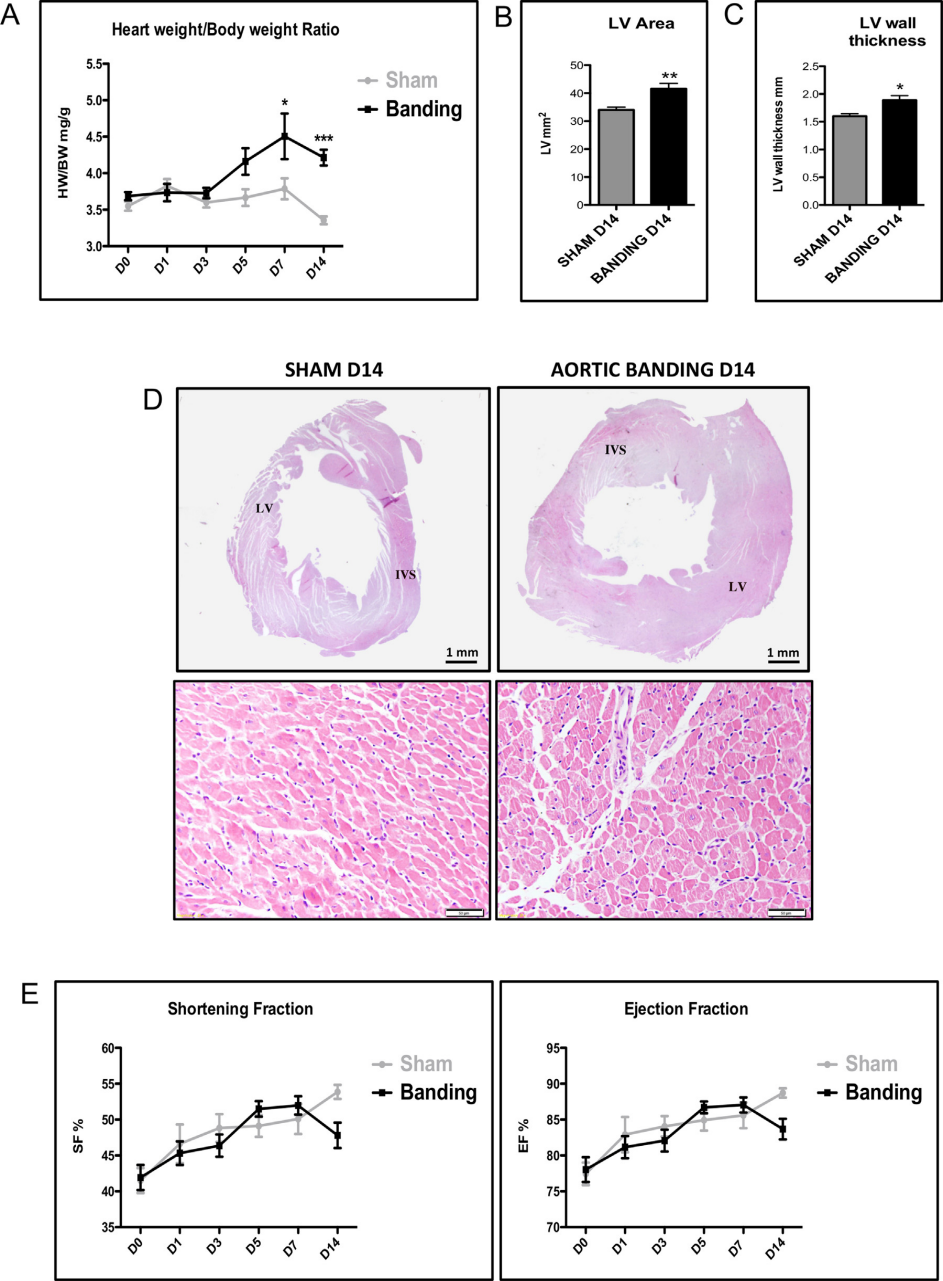
Left ventricle anatomy and function in rat LVH. (A) Heart (left and right ventricles) to body weight ratio (mg/g) in aortic-banded (black line) *vs* sham-operated animals (gray line) from D0 to D14 (n = 6 per group). (B) Left ventricle area in sham-operated (gray block) *vs* aortic-banded (black block) animals (n = 6 per group) at D14. (C) Left-ventricle wall thickness in aortic banded *vs* sham-operated groups (n = 6 per group). (D) Hematoxylin-eosin staining of left-ventricular sections from sham operation (left) *vs* aortic banding (right) samples at D14. LV: Left ventricle. IVS: Interventricular septum. (E) Echocardiography measurements of the shortening and ejection fractions, calculated as: FS = [(EDLVD-ESLVD)/EDLVD)] x100; EF = [(EDLVD^3^-ESLVD^3^) / EDLVD^3^] x100. The end-diastolic and end-systolic left ventricle diameters (EDLVD and ESLVD) were measured on parasternal short-axis view of LV from M-mode. Data are presented as means ± SEM. *P<0.05 **P<0.01 ***P<0.001 compared to sham group.

In contrast to the expression of inflammatory genes that peaked at D3 or D5, the expression of ANP mRNA the endogenous hypertrophic marker, increased progressively in the aortic-banded *vs* sham-operated groups, with statistically significantly higher levels at D5 (50±24-fold, *P*<0.001), decreasing at D7 (34±20-fold, *P*<0.01) and D14 (18±9-fold, *P*<0.01) (Fig 1B). BNP mRNA expression, another positive control for cardiac hypertrophy, also increased significantly in the aortic-banded *vs* sham-operated groups, with statistically higher levels at D3 (7.3±2.9-fold, *P*<0.001), at D5 (3.6±1.1-fold, *P*<0.05), at D7 (3.2±0.6-fold, *P*<0.05) and at D14 (3.7±1-fold, *P*<0.01) (Fig 1B). The manifestation of hypertrophy at this later phase was confirmed macroscopically by the heart weight/body weight ratio that increased progressively over time in the aortic-banded *vs* the sham-operated group (Fig 2A): it was significantly higher at D7 (15.9% increase, *P*<0.05) and D14 (20.4% increase, *P*<0.001). LVH at this later stage was visualized by histological analysis of the LV wall area and thickness, which were significantly larger in the aortic-banded (41.6±1.9 mm^2^ and 1.9±0.1 mm, respectively) than the sham-operated animals (34.0±1.0 mm^2^ and 1.6±0.1 mm, *P*<0.05 for both; Fig 2B and 2C). Fig 2D presents the hematoxylin-eosin staining of LV sections from sham-operated and aortic-banded animals at D14, and S3 Fig at D0-D7. Data for LV wall area and thickness over time are presented in S2 Table. Echocardiographic values of the interventricular septum and posterior wall thickness of the LV in diastole at D14 confirmed significantly higher measurements in the aortic-banded (1.24±0.03 and 1.12±0.02 mm, respectively) *vs* sham-operated groups (0.97±0.02 and 0.83±0.02 mm) (*P*<0.001 for both, S4 Fig). The results indicate concentric LVH in this model. None of the animals developed cardiac dysfunction, as the shortening (FS) and ejection fractions (EF) did not differ significantly between the two groups and remained within the normal range (FS > 40% and EF > 75%) (Fig 2E). These findings suggest that the hypertrophy is compensated throughout the entire 14 days study period where the left ventricle function is still preserved.

### Expression of chemokine mRNAs in rat LVH

**Fig 3:**
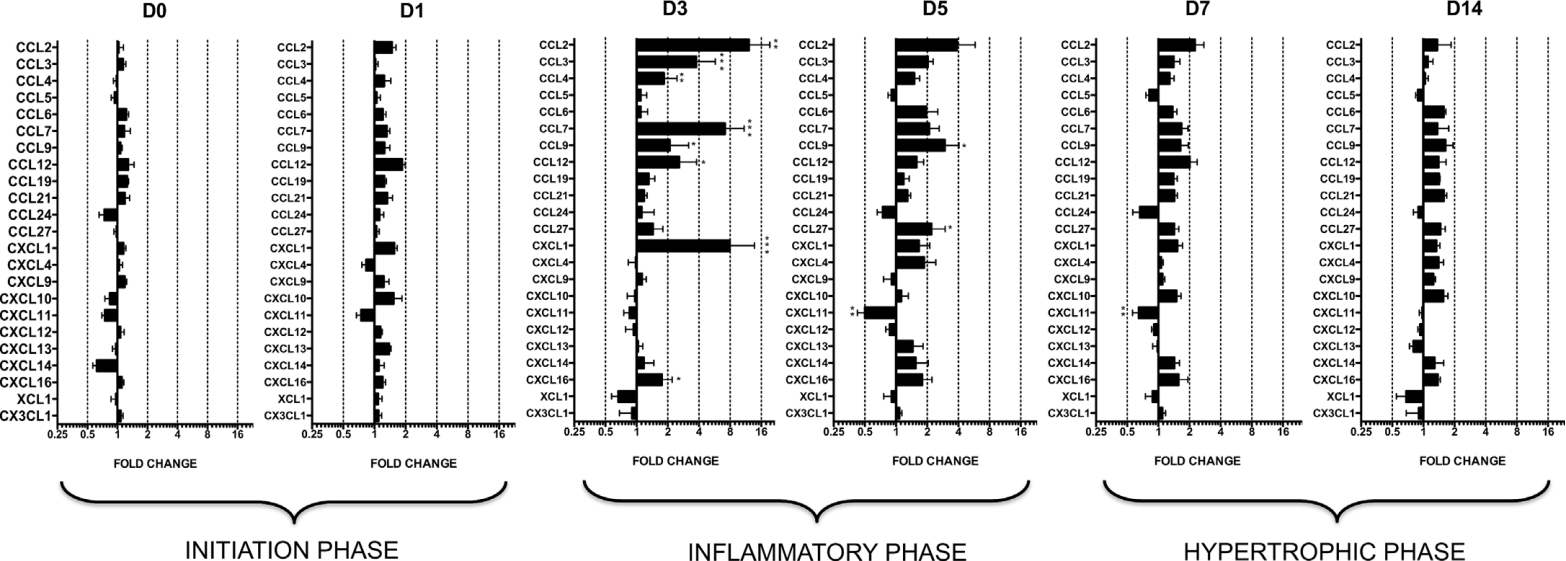
Relative mRNA expression levels of chemokines in aortic banding groups at D0, D1, D3, D5, D7, and D14. Fold change is calculated as 2^(-ΔΔCt)^ where the calibrator is the mRNA level of the gene in the sham group. Only genes for which Ct is <32 are presented. For each sample, two qPCRs were performed on different parts of the LV, one from the apex, and another from the base part. Means are calculated for each animal (n = 6 per group). Fold change levels are represented on a logarithmic scale. Data are means (blocks) ± SEM (bars) of n = 6 animals.

Of the 36 chemokine genes studied, 13 chemokines were not expressed in any animals at any time point (Ct>32) (S1 Table). The 23 mRNAs that were expressed (Ct<32) were analyzed and compared over time (D0, D1, D3, D5, D7, and D14) between the two groups (Fig 3). First, the excellent reproducibility of the chemokine mRNA levels in the sham-operated animals over time allowed us to compare them with the aortic-banded rats. The global analysis showed that the mean fold differences between the groups peaked at D3 (2.4±0.6) and D5 (1.6±0.2), while chemokine levels remained relatively constant and similar at D0 (1.0±0.1), D1 (1.2±0.1), D7 (1.3±0.1), and D14 (1.2±0.1) (S5A Fig). This indicates that overexpression of chemokines in the aortic-banded animals occurred at D3 and D5 post-surgery and normalized thereafter even as LVH progressed (D7-D14). The absence of overexpression of any chemokine (Fig 3) or inflammatory gene (Fig 1) at D14, the end point, indicates that their expression remained regulated during the compensated phase of LVH.

**Fig 4:**
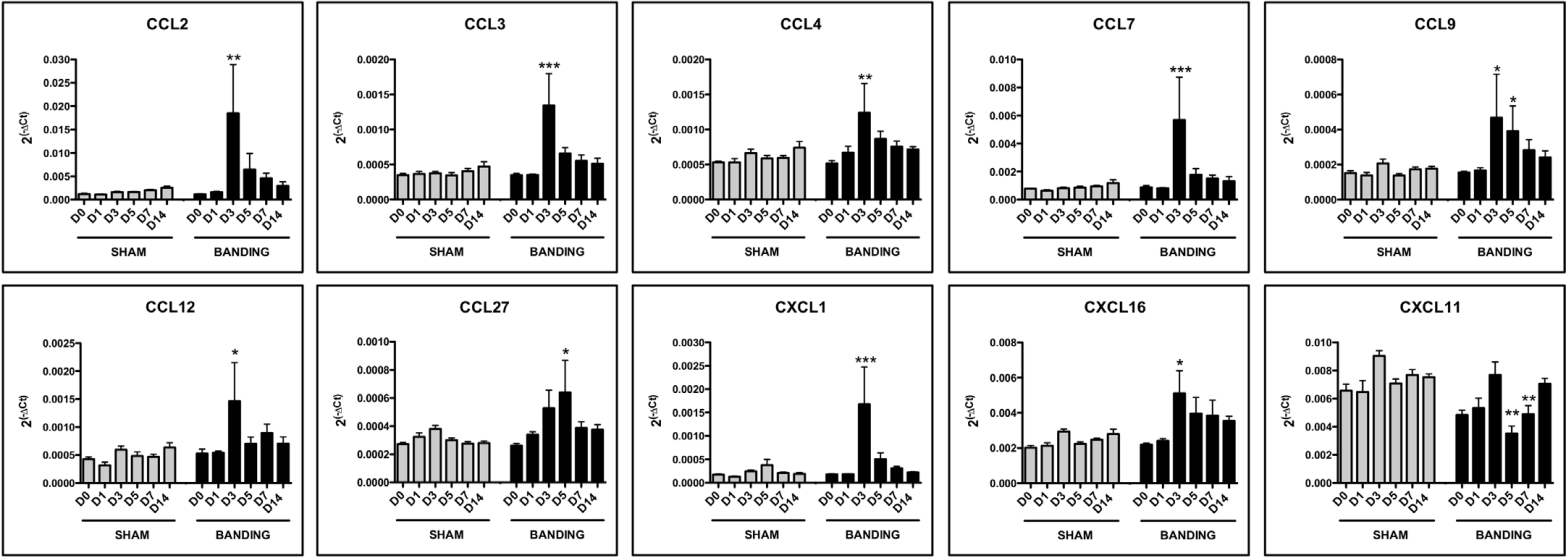
mRNA overexpression of chemokines in aortic-banded *vs* sham-operated groups. Expression is calculated as 2^(-ΔCt)^ where the calibrator is the mRNA level of the *gapdh* reference gene. Data are presented as means (blocks) ± SEM (bars). *P<0.05 **P<0.01 ***P<0.001 compared to sham group.

Fig 4 shows a selection of the overexpressed chemokines (2^-ΔCt^ values) in the aortic-banded vs. sham-operated groups. CCL2 (12.2±7.2-fold, *P*<0.01), CCL7 (7.1±3.8-fold, *p*<0.001), CCL3 (3.8±1.9-fold, *P*<0.001), CXCL1 (7.9±5.7-fold, *P*<0.001), CCL12 (2.6±1.2-fold, *P*<0.05), CCL4 (1.8±0.6-fold, *P*<0.01) and CXCL16 (1.8±0.4-fold, *P*<0.05) were significantly overexpressed at D3, but decreased thereafter. Overexpression of CCL9 continued longer: at D3 (2.0±1.0-fold, *P*<0.05) and D5 (3.0±1.1-fold, *P*<0.05), but recovered slightly, returning to baseline levels in both groups. CCL27 overexpression occurred later on, 2.2±0.8-fold higher on D5 (*P*<0.05) after more scattered levels on D3 (Fig 4). Two chemokines showed significantly higher levels at D14 in the aortic-banded group (Fig 3), although the fold difference did not exceed 1.5: CCL19 with 1.4±0.1-fold (*P*<0.05) and CCL21 with 1.5±0.1-fold (*P*<0.05). Only one gene in the entire study, *cxcl11*, showed downregulation—slight but significant—in the aortic-banded compared to sham-operated animals, with a 0.5±0.1-fold decrease at D3 (*P*<0.01) and 0.7±0.1-fold at D5 (*P*<0.01) (Fig 4).

### Expression of chemokine receptor mRNAs in rat LVH

**Fig 5:**
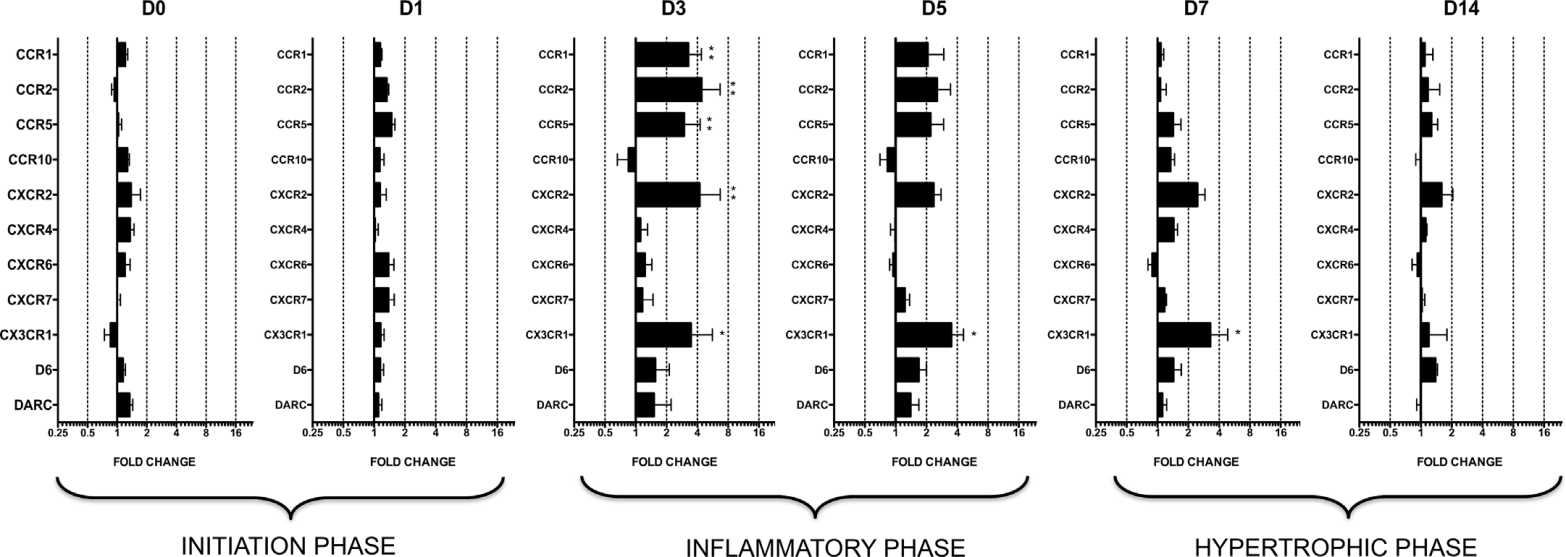
Relative mRNA expression levels of chemokine receptors in aortic banding groups at D0, D1, D3, D5, D7, and D14. Fold change is calculated as 2^(-ΔΔCt)^ where the calibrator is the mRNA level of the gene in the sham group. Only genes for which the Ct is <32 are presented. For each sample, two qPCRs are performed on different parts of the LV, one from the apex, and another from the base part. Means are calculated for each animal (n = 6 per group). Fold change levels are represented on a logarithmic scale. Data are means (blocks) ± SEM (bars) of n = 6 animals.

Nine of the 20 chemokine receptors studied were not expressed in any animals at any point (Ct>32) (S1 Table). The global analysis of chemokine receptors showed that the mean fold difference between the aortic-banded and sham-operated groups peaked at D3 (2.3±0.4) and D5 (1.8±0.2), but remained relatively constant and similar at D0 (1.2±0.4), D1 (1.2±01), D7 (1.5±0.2), and D14 (1.2±0.1) (S5B Fig). This profile is thus similar to that for chemokine expression: the highest overexpression took place during the inflammatory phase (D3-D5), before the presence of LVH (Fig 5).

**Fig 6:**
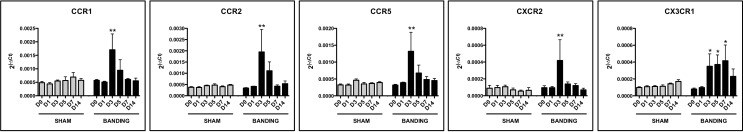
mRNA overexpression of chemokine receptors in aortic-banded *vs* sham-operated groups. Expression is calculated as 2^(-ΔCt)^ where the calibrator is the mRNA level of the *gapdh* reference gene. Data are represented as means (blocks) ± SEM (bars). *P<0.05 **P<0.01 compared to sham group.

Levels of CCR1 (3.3±1.1-fold, *P*<0.01), CCR2 (4.4±2.3-fold, *P*<0.01), CCR5 (3.0±1.3-fold, *P*<0.01) and CXCR2 (4.2±2.5-fold, *P*<0.01) were significantly higher at D3 in the aortic-banded as compared to sham-operated groups and decreased thereafter (Fig 6). Only CX3CR1 was significantly overexpressed over time in the banded group, at levels more than three times higher than in the sham-operated animals from D3 through D7: D3 (3.5±2.2-fold, *P*<0.05), D5 (3.5±1.1-fold, *P*<0.05), and D7 (3.3±1.6-fold, *P*<0.05), with normalized expression thereafter (D14) (Fig 6).

## Discussion

Our study reports the first exhaustive analysis of the kinetics of chemokine and chemokine receptor mRNA expression during the development of LVH in the rat. The aortic-banded rat model is one of the most reliable and reproducible models of LVH: aortic constriction leads to pressure overload and to a left-ventricular hypertrophic phenotype detectable from D7. Our kinetic study reports analyses on D0, D1, D3, D5, D7, and D14 in both groups. Results clearly show the presence of an early inflammatory phase in the first 3–5 days after aortic banding, characterized by increased mRNA expression of IL-1ß, TNFa, NLRP3, and the RelA subunit of NF-kB. This inflammatory phase is associated with overexpression of chemokines mRNA (CCL2, CCL3, CCL4, CCL7, CCL9, and CCL12, and CXCL1 and CXCL16) and of chemokine receptors (CCR1, CCR2, CCR5, CXCR2, CX3CR1). The inflammatory phase is followed by the hypertrophy phase (D7-D14) characterized by ANP and BNP mRNA overexpression, during which time chemokine and receptor expression return to baseline levels (D0). This phase coincides with the physiological compensated phase of hypertrophy in our model, as seen from the echocardiographic measurements.

Among the panel of genes found to be overexpressed during the inflammatory phase in our model, are the monocyte chemoattractant proteins MCP1, 3, and 5 (*ccl2*, *7*, and *12*), together with the macrophage inflammatory proteins MIP1a, ß, and g (*ccl3*, *4*, and *9*). Their primary function is the recruitment to the inflammation site of monocytes/macrophages, expressing their respective chemokine receptors (CCR1, 2, 3, 5) [36–38]. CCL2/MCP1 is the chemokine that has been studied most often in the hypertrophic heart. Elevated levels are found in the cardiac biopsies of patients with aortic stenosis, correlated with newly recruited leukocytes [16]. Fibrosis in CCL2 KO mice is attenuated at D5 and D7 in an ischemic cardiomyopathy model developed by intermittent daily induction of ischemia/reperfusion [22]. In parallel, invalidation of CCL2 by gene therapy in a murine model of left-ventricular remodeling and failure improves survival rates and inhibits the cardiac failure characteristics associated with interstitial fibrosis and macrophage infiltration [39]. CCL2 neutralization by anti-CCL2 blocking antibody also leads to decreased cardiac perivascular and interstitial fibrosis, which is associated with LVH, but does not decrease the cardiomyocyte hypertrophy itself [40]. Overexpression of CCL2 in cardiac myocytes in alpha-cardiac myosin heavy chain (MHC)-MCP1 mice increases cardiac monocyte recruitment and left-ventricular dysfunction [41, 42]. Consistently with these observations, in our study CCL2 mRNA was the top overexpressed chemokine—by about 12-fold—in the aortic-banded animals, together with CCR2, the major CCL2 receptor. Our data encourage the investigation of the blocking effect of CCL2/CCR2 action on development of LVH.

Levels of another macrophage-attracting chemokine, CCL12/MCP-5, which also interacts with CCR2, increased at D3 in the aortic-banded animals. CCL12 is described as a chemotactic chemokine for inflammatory cells, such as the macrophages and fibroblast precursors expressed during lung inflammation and infection [43–45]. To our knowledge, this study is the first to demonstrate CCL12 mRNA expression in cardiac hypertrophy.

CCL7/MCP-3, another chemokine from the MCP family, and its receptors, CCR1 and CCR2, were also overexpressed during the inflammatory phase in the aortic-banded animals in our LVH model. The presence of this chemokine has not previously been reported in cardiac hypertrophy. One of its primary functions is to mobilize monocytes from the bone marrow (along with CCL2 activating CCR2) and promote their migration to the inflamed tissue, but it is also critical for maintaining leukocyte homeostasis in normal conditions [46].

The macrophage inflammatory protein family mRNA was also significantly overexpressed in the aortic-banded animals. CCL9/MIP1g, a ligand for CCR1, was overexpressed at both D3 and D5. Like the other members of the MIP chemokine family, CCL9 is chemotactic for inflammatory monocytes [47, 48], but it has not previously been reported in LVH. CCL3/MIP1a is inducible in a number of hematopoietic cells, particularly in those involved in adaptive immune responses (macrophages, dendritic cells, and B and T lymphocytes). Little information is thus far available about its involvement in LVH, but its expression is upregulated in serum from patients with heart failure [24]. CCL3 activates CCR1 and CCR5, both of which were three times higher at D3 in the banded rats. Both receptors also interact with CCL4/MIP1ß, which was slightly but significantly overexpressed (1.8-fold) in aortic-banded hearts. This finding is in line with data from patients with aortic stenosis: CCL4 has been found to be overexpressed in the myocardium of their hypertrophic hearts [16].

In our model, peak expression of chemokine mRNA from the MCP and MIP families coincided with the timing of macrophage infiltration in left ventricles at D3 and D6, previously reported in murine models of LVH [14, 23], and thus confirmed the inflammatory events preceding hypertrophy. Studies report the presence of both M1 and M2 macrophages in cardiac hypertrophy: the proinflammatory M1 macrophages express miR-155 microRNA and thereby increase the hypertrophic phenotype of cardiac tissue, while M2 macrophages are considered to be cardioprotective [14, 49, 50]. These findings are consistent with the hypothesis of that the M1/M2 polarization status of cardiac macrophages affects hypertrophic compensatory mechanisms and adaptive responses [50].

Finally, and surprisingly, we found significant overexpression of the cutaneous CCL27 chemokine mRNA at D5 in the aortic-banded groups, although expression of its receptor CCR10 did not change. CCL27 is known to recruit lymphocytes to cutaneous wounds [51]; it has not yet been demonstrated to play a role in any cardiac diseases. Further studies are required to clarify the function of this overexpression.

Expression of a few CXC chemokine mRNA also changed over time in the aortic-banding model. CXCL1 was overexpressed at D3, together with its receptor CXCR2. CXCL1 (GRO1, growth-regulated oncogene 1) is a chemoattractant for neutrophils, T lymphocytes, and monocytes; it induces free radical production and thus leads to endothelial damage [52].

Another interesting group of chemokines are CXCL16 and CX3CL1 (fractalkine), both synthesized as transmembrane multidomain proteins composed of a chemokine domain, a glycosylated mucin-like stalk, and a single transmembrane helix [53]. Both chemokines can act as adhesion molecules for leukocytes as well as soluble chemokines after cleavage by ADAM (A Desintegrin And Metalloproteinase). In this study, CXCL16 mRNA was significantly overexpressed at D3. Its *in vitro* promotion of myocardial fibroblast proliferation and its augmentation of matrix metalloproteinase activity in cardiomyocytes cause it to play an important role in hypertrophy [27]. CXCL16 and CX3CL1 together regulate expression and posttranslational modifications of the small leucine-rich proteoglycans that regulate the extracellular matrix in cultured cardiac fibroblasts [28]. Consistent with this observation, CXCL16 overexpression occurs in the left-ventricular tissue of aortic-banded mice, and its upregulation is reported in the plasma of patients with right-ventricular hypertrophy due to pulmonary valve stenosis and heart failure; its expression levels are correlated with the severity of heart failure [28]. Moreover, studies of CXCR6 KO mice have shown that the CXCR6/CXCL16 pair plays an essential role in the pressure overload-mediated recruitment of monocytes that contributes to cardiac fibrosis [54]. In our model, however, mRNA expression of CXCR6 did not differ from that of the sham-operated (control) left ventricles, nor was CX3CL1 expression in LVH modified. CX3CL1 expression increases in the heart and serum of patients with end-stage heart failure due to coronary artery disease or dilated cardiomyopathy [29], which involves however different mechanisms from the compensated hypertrophy due to arterial hypertension or aortic stenosis. In a mouse model of cardiac remodeling induced by transverse aortic constriction, administration of an anti-CX3CL1 neutralizing antibody significantly slows hypertrophy progression [26]. In our study, expression of CX3CR1, the CX3CL1 receptor, was higher in the aortic-banded group on D3, D5, and D7, *i*.*e*. in the inflammatory phase and early in the hypertrophic phase. It was thus the longest lasting receptor mRNA overexpression in our study. CX3CR1 is reported to distinguish between monocyte/macrophage populations [14]. These authors have suggested that increased expression of CX3CR1 at the cell surface of the anti-inflammatory M2 macrophages might further promote their adhesion to the endothelium-expressing membrane CX3CL1. Targeting the CX3CR1 receptor might thus contribute to decreasing the accumulation of inflammatory cells in the left ventricle, which progresses to fibrosis.

In our LVH model, neither CXCL12 mRNA (SDF-1a, Stromal cell-derived factor 1a) nor its receptor CXCR4 was expressed. This finding contrasts with the upregulation of CXCL12 and CXCR4, respectively in the plasma and left ventricles, in patients with chronic heart failure due to coronary artery disease or dilated cardiomyopathy and in two mouse models of cardiac hypertrophy, one due to transaortic constriction and the other using transgenic RacET mice with cardiac overexpression of the small GTPase Rac1 [32].

Finally, the only downregulated gene we found in our study was *cxcl11*, an IFNg-inducible chemokine [55]. No IFNg expression was observed in our model. Little information is available on this chemokine’s potential role in cardiac disease. Elevated serum levels are associated with the development of coronary artery disease after heart transplantation [56] and with the progression from asymptomatic to symptomatic diastolic left ventricular dysfunction in hypertensive patients [57]. It will be interesting to analyze the expression of CXCL11 in LV later in our model, during development of the cardiac decompensation. The role of CXCL11 and its downregulation in the early phase of rat LVH model requires further investigation.

We must underline that the LVH studied during the observation period in our model was in its compensated stage, as shown by the echocardiographic analysis, which detected no cardiac dysfunction. Mice with inadequate cardiac hypertrophic response show early heart failure and die earlier [58]. Chemokines are thought to act directly on resident cardiomyocytes to induce cardioprotective mechanisms, as reported for CCL2 overexpression, which protects cultured cardiomyocytes from hypoxia-induced cell death [59, 60]. Likewise, the association between the overexpression of CCL2/MCP1 in MHC-MCP1 mice and improved cardiac tissue repair after myocardial infarction suggests that this chemokine plays a role in tissue remodeling after damage [61].

The information provided by our study is important for understanding the development of the inflammatory phase of cardiac hypertrophy as well as the chemokine/receptor interactions. The protein expression of these chemokines and receptors together with the presence of infiltrated immune and inflammatory cells will add new insights into the role of these chemokines in LVH. Moreover, an analysis of expression of chemokines and receptors in a failing heart model, i.e. during the transition from compensated to decompensated LVH, would therefore be useful for delineating their roles in both stages of hypertrophy.

In conclusion, our rat model of LVH without cardiac dysfunction exhibited an inflammatory phase at D3-D5 after aortic banding and then evolved into a phase with a clear hypertrophic phenotype at D7-D14. Comprehensive analysis of chemokine and receptor mRNA expression revealed overexpression during the inflammatory phase, followed by a return to baseline levels in the hypertrophic phase. Most of the overexpressed chemokines belong to the monocyte/macrophage chemotactic families MIP and MCP. Further elucidation of the role of these chemokines in the inflammatory response (recruitment of proinflammatory cells) and/or hypertrophic compensatory mechanisms (protection of resident cardiomyocytes) is now necessary to understand whether targeting the chemokine network during pressure overload might usefully supplement the blockage of the hypertrophic signaling pathways currently available in clinical settings.

## Supporting Information

S1 Fig Housekeeping gene mRNA expression. Ct values of housekeeping genes are displayed for all sham-operated (left) and aortic banding (right) samples. The stability of the expression of each gene is calculated by the DataAssist® Software and given as stability score. The lower the score, the more stable the expression of the reference gene is [33]. Ct = threshold cycle.
(TIFF)

S2 Fig SCF mRNA expression in rat LVH. mRNA expression of SCF in aortic-banded *vs* sham-operated groups (n = 6 per group). Expression is calculated as 2^(-ΔCt)^ where the calibrator is the mRNA level of the *gapdh* reference gene. Data are presented as mean ± SEM.
(TIFF)

S3 Fig LV anatomy D0-D7. Hematoxylin-eosin staining of left ventricular sections from sham (left) *vs* aortic banding (right) samples at D0-D7. LV: Left ventricle. IVS: Interventricular septum.
(TIFF)

S4 Fig Echocardiographic assessment of cardiac anatomy. Interventricular septum and posterior wall thickness of LV in diastole were measured on the parasternal short-axis view of LV. ***P<0.001 compared to sham group.
(TIFF)

S5 Fig Global fold change analysis of mRNA expression. Mean fold change differences of aortic-banding *vs* sham groups from D0 to D14 for chemokines (A) and chemokine receptors (B). **P<0.01 ***P<0.001 compared to sham group.
(TIFF)

S1 Table List of analyzed genes and primers. List of the primers used for the design of mRNA expression array. The Applied Biosystems® reference number of the Taqman® primers is given for each gene. In italic are shown the genes for which the Ct value was higher than 32 cycles and which were then not analyzed. Analyzed genes are presented in bold. Ct = threshold cycle.
(PDF)

S2 Table Histological analysis of LV. Measurements of the left ventricle area and left ventricle wall thickness in sham-operated and aortic-banded animals from D0 to D14 (n = 6 per group). Analysis was performed with the ImageJ software on slides after hematoxylin-eosin staining.
(PDF)

## Abbreviations

ADAM: A Desintegrin and Metalloproteinase
ANP: Atrial natriuretic peptide
cDNA: Complementary Deoxyribonucleic Acid
CRP: C-Reactive Protein
EDLVD: End-Diastolic Left Ventricle Diameters
EF: Ejection Fraction
ESLVD: End-Systolic Left Ventricle Diameters
GAPDH: Glyceraldehyde-3-Phosphate Dehydrogenase
GPCR: G protein-Coupled Receptors
GRO1: Growth-Regulated Oncogene 1
HMBS: Hydroxyl-Methylbilane Synthase
HPRT1: Hypoxanthine Phosphoribosyltransferase
IFNγ: Interferon γ
IL: Interleukin
LV: Left Ventricle
LVH: Left-Ventricular Hypertrophy
MCP: Monocyte Chemoattractant Protein
MIP: Macrophage Inflammatory Protein
mRNA: Messenger Ribonucleic Acid
NF-κB: Nuclear Factor-Kappa B
NLRP3: NOD-like Receptor Family, Pyrin Domain Containing 3
SCF: Stem Cell Factor
SDF: Stromal Cell-derived Factor
FS: Fractional Shortening
TBP: TATA Box Binding Protein
TGF-β: Transforming Growth Factor β
TNFα: Tumor Necrosis Factor α
