# Supplementary figures and images for: Kinetic mRNA Profiling in a Rat Model of Left-Ventricular Hypertrophy Reveals Early Expression of Chemokines and Their Receptors
Source: PLoS One. 2016 Aug 15;11(8):e0161273. doi: 10.1371/journal.pone.0161273 (PMC4985150; doi:10.1371/journal.pone.0161273)

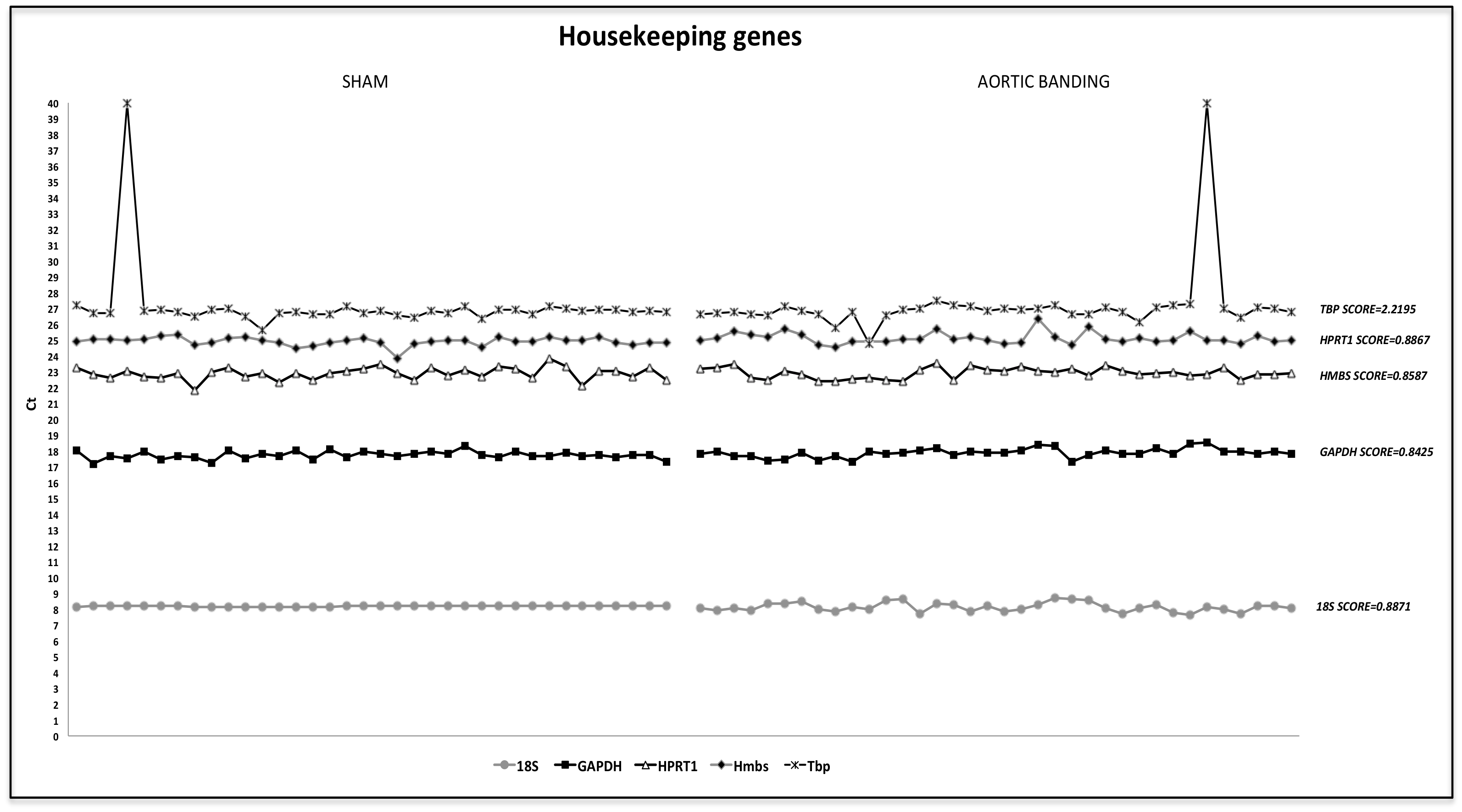

Supplement: S1 Fig — Ct values of housekeeping genes are displayed for all sham-operated (left) and aortic banding (right) samples. The stability of the expression of each gene is calculated by the DataAssist® Software and given as stability score. The lower the score, the more stable the expression of the reference gene is [33]. Ct = threshold cycle. (TIFF) [file pone.0161273.s001.tiff]

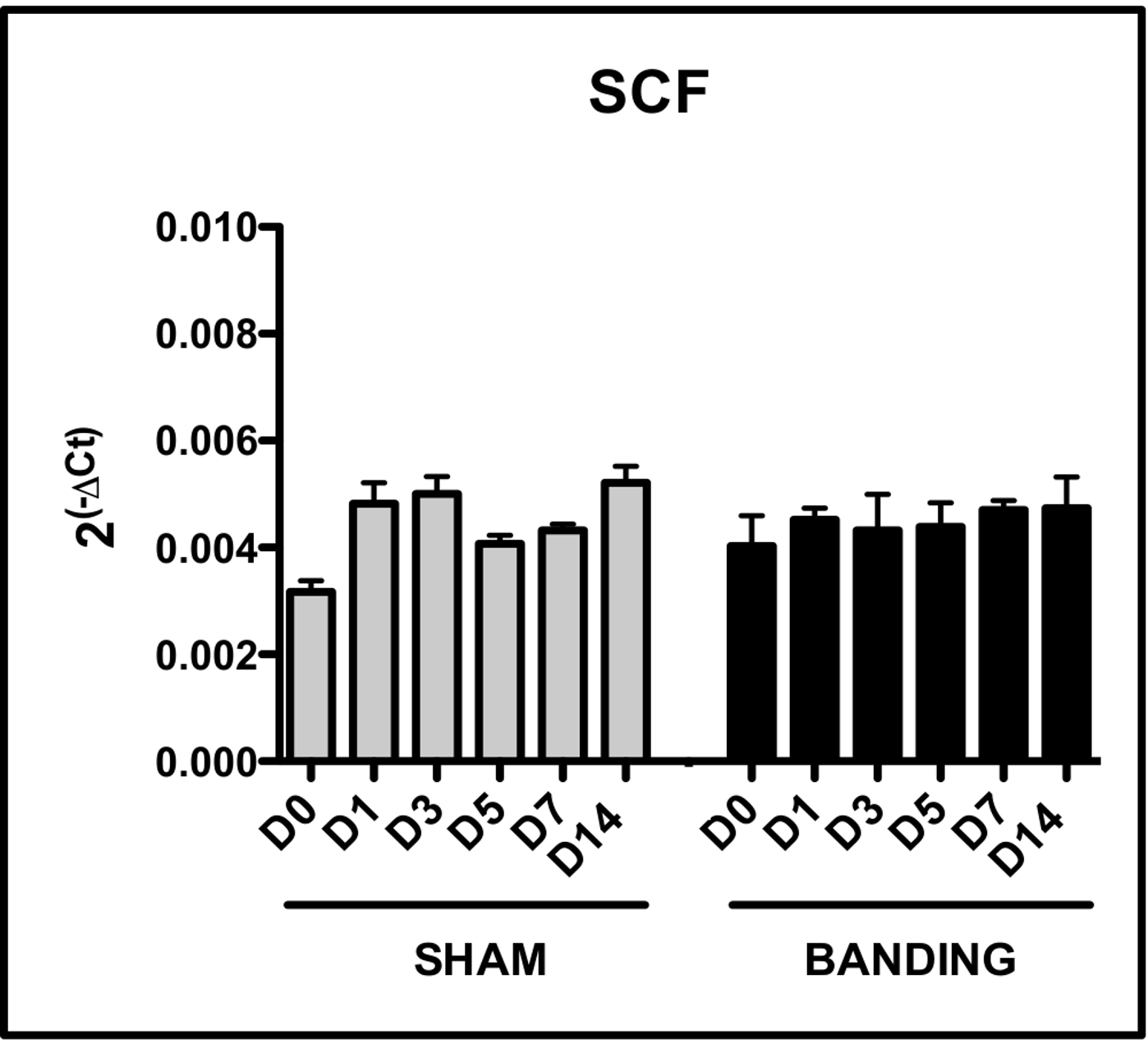

Supplement: S2 Fig — mRNA expression of SCF in aortic-banded vs sham-operated groups (n = 6 per group). Expression is calculated as 2(-ΔCt) where the calibrator is the mRNA level of the gapdh reference gene. Data are presented as mean ± SEM. (TIFF) [file pone.0161273.s002.tiff]

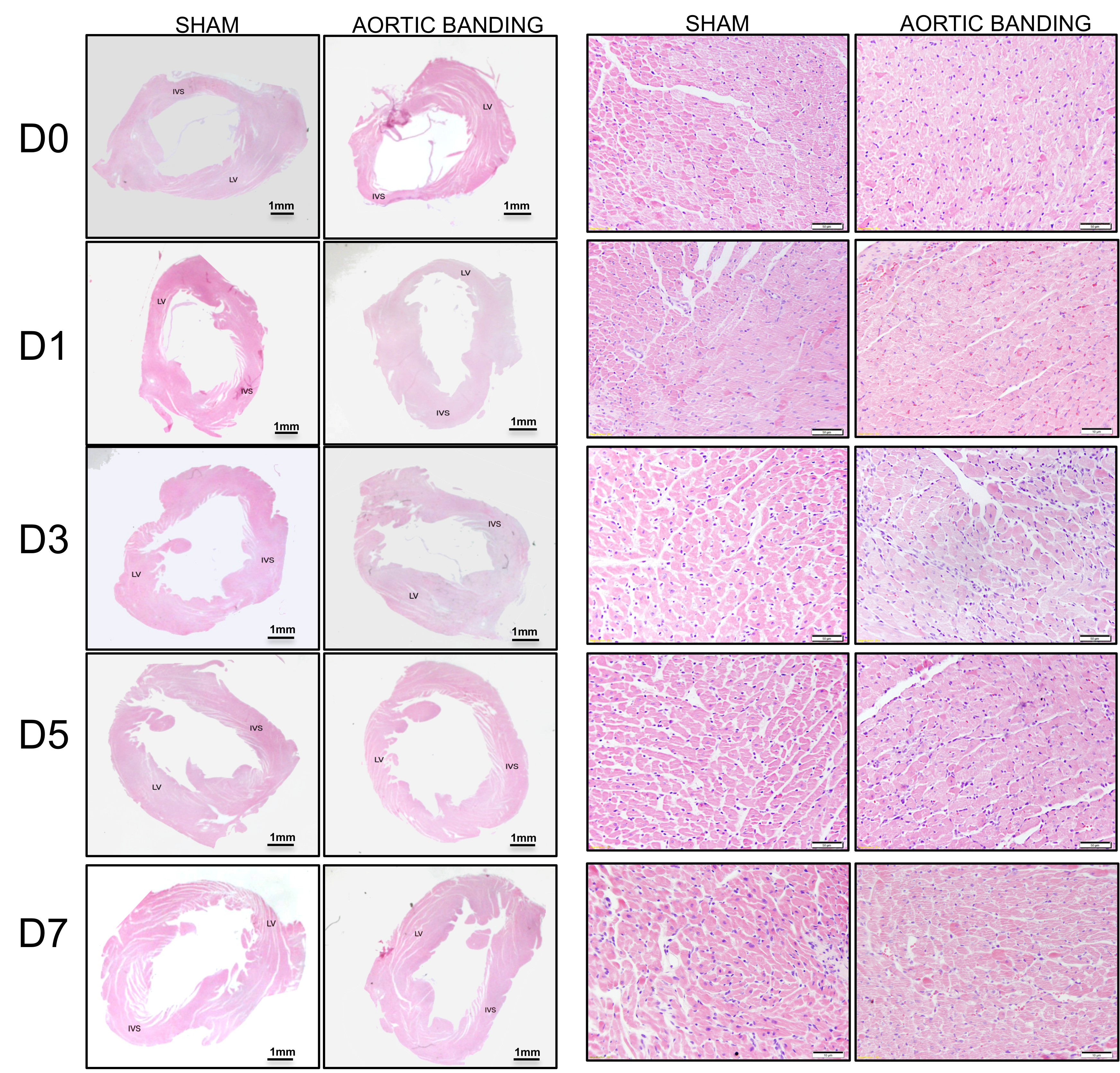

Supplement: S3 Fig — Hematoxylin-eosin staining of left ventricular sections from sham (left) vs aortic banding (right) samples at D0-D7. LV: Left ventricle. IVS: Interventricular septum. (TIFF) [file pone.0161273.s003.tiff]

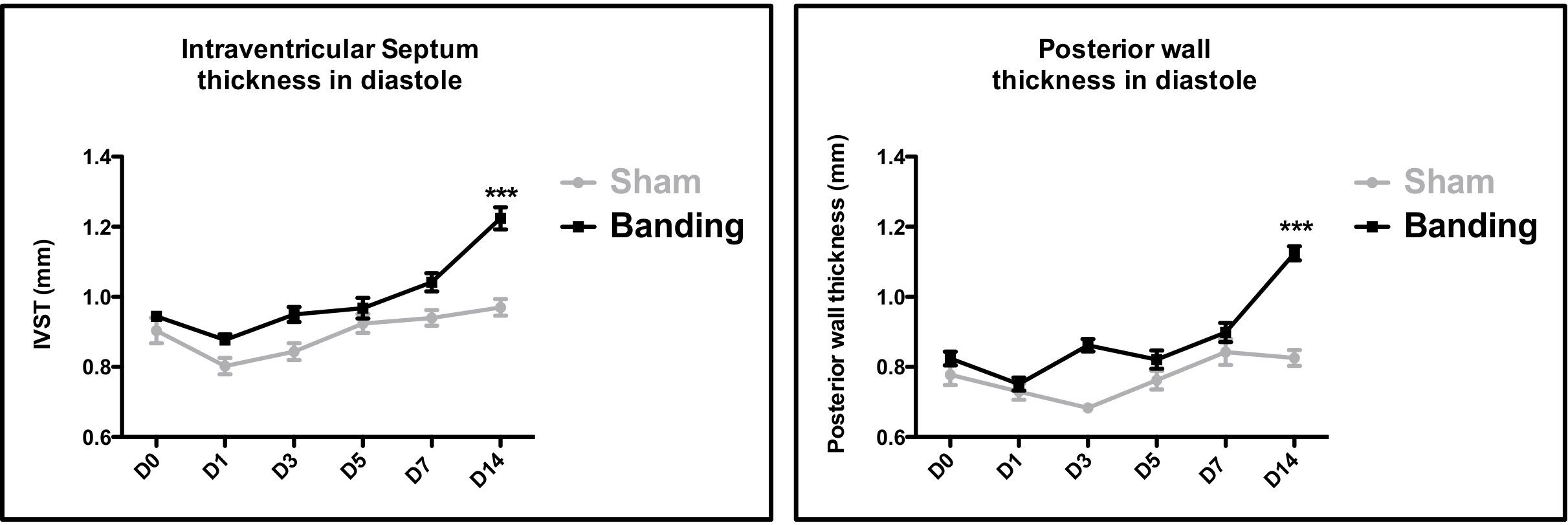

Supplement: S4 Fig — Interventricular septum and posterior wall thickness of LV in diastole were measured on the parasternal short-axis view of LV. ***P<0.001 compared to sham group. (TIFF) [file pone.0161273.s004.tiff]

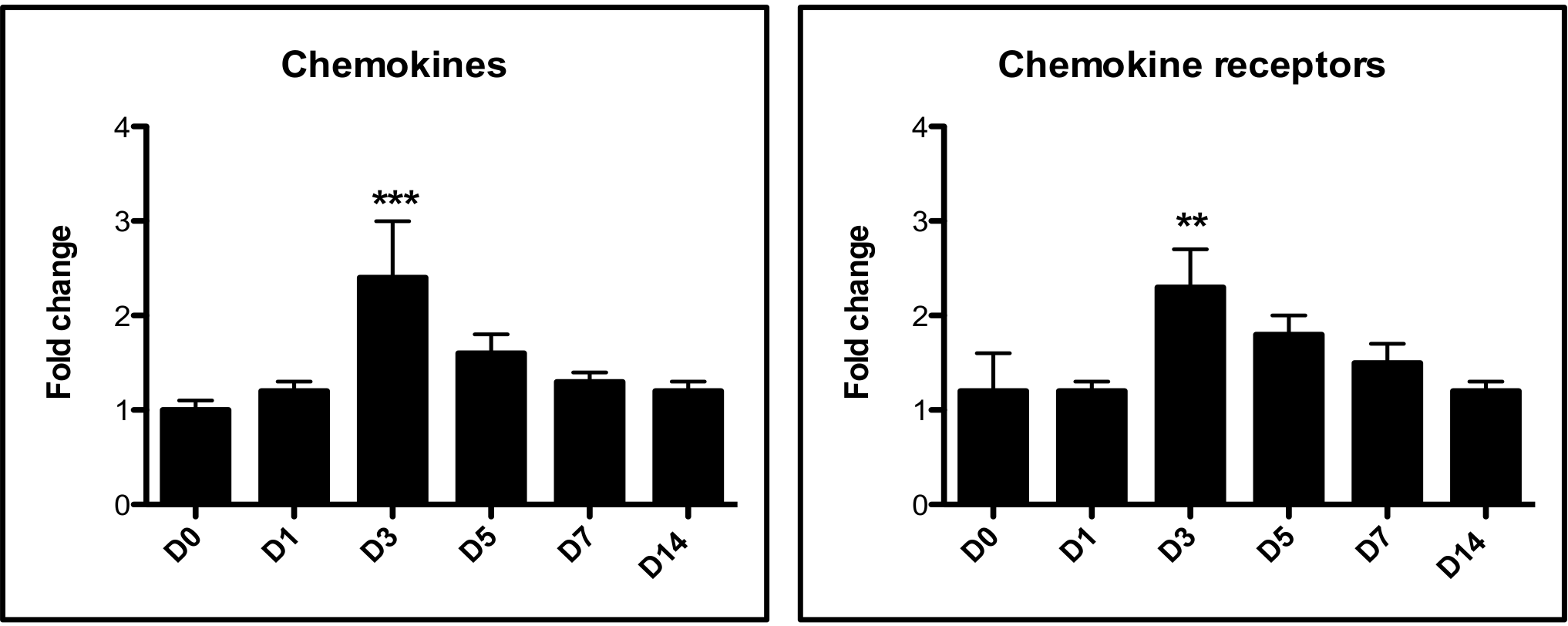

Supplement: S5 Fig — Mean fold change differences of aortic-banding vs sham groups from D0 to D14 for chemokines (A) and chemokine receptors (B). **P<0.01 ***P<0.001 compared to sham group. (TIFF) [file pone.0161273.s005.tiff]
