# Supplementary material for: Kinetic mRNA Profiling in a Rat Model of Left-Ventricular Hypertrophy Reveals Early Expression of Chemokines and Their Receptors
Source: PLoS One. 2016 Aug 15;11(8):e0161273. doi: 10.1371/journal.pone.0161273 (PMC4985150; doi:10.1371/journal.pone.0161273)
Supplement: S1 Table — List of the primers used for the design of mRNA expression array. The Applied Biosystems® reference number of the Taqman® primers is given for each gene. In italic are shown the genes for which the Ct value was higher than 32 cycles and which were then not analyzed. Analyzed genes are presented in bold. Ct = threshold cycle. (PDF) [file pone.0161273.s006.pdf]

Chemokines			Chemokine receptors			Inflammatory genes		
CCL1	*Rn01752376_m1*	*Ct>32*	**CCR1**	**Rn00571950_s1**		*CRP*	*Rn00567307_g1*	*Ct>32*
**CCL2**	**Rn00580555_m1**		**CCR2**	**Rn01637698_s1**		**IL1β**	**Rn00580432_m1**	
**CCL3**	**Rn01464736_g1**		*CCR3*	*Rn02134292_s1*	*Ct>32*	*IL6*	*Rn01410330_m1*	*Ct>32*
**CCL4**	**Rn00671924_m1**		*CCR4*	*Rn00591783_s1*	*Ct>32*	*IL17A*	*Rn01757168_m1*	*Ct>32*
**CCL5**	**Rn00579590_m1**		**CCR5**	**Rn02132969_s1**		*IFNγ*	*Rn00594078_m1*	*Ct>32*
**CCL6**	**Rn01456400_m1**		*CCR6*	*Rn02534497_s1*	*Ct>32*	**TNFα**	**Rn01525859_g1**	
**CCL7**	**Rn01467286_m1**		*CCR7*	*Rn02758813_s1*	*Ct>32*	**NLRP3**	**Rn04244625_m1**	
**CCL9**	**Rn01471276_m1**		*CCR9*	*Rn02129940_s1*	*Ct>32*	**SCF**	**Rn01510586_g1**	
CCL11	*Rn00569995_m1*	*Ct>32*	*CCR10*	*Rn03037167_s1*	*Ct>32*	**RelA**	**Rn01483598_m1**	
**CCL12**	**Rn01464638_m1**		*CXCR1*	*Rn00570857_s1*	*Ct>32*			
CCL17	*Rn01536936_g1*	*Ct>32*	**CXCR2**	**Rn02130551_s1**				
**CCL19**	**Rn01439563_m1**		*CXCR3*	*Rn02134090_s1*	*Ct>32*			
CCL20	*Rn00570287_m1*	*Ct>32*	**CXCR4**	**Rn00573522_s1**				
**CCL21**	**Rn01764651_g1**		*CXCR5*	*Rn02132880_s1*	*Ct>32*			
CCL22	*Rn01536591_m1*	*Ct>32*	**CXCR6**	**Rn03037244_s1**				
**CCL24**	**Rn01481451_m1**		**CXCR7**	**Rn02134053_s1**				
CCL25	*Rn01403352_m1*	*Ct>32*	*XCR1*	*Rn03037149_s1*	*Ct>32*			
CCL26	*Rn01481484_m1*	*Ct>32*	**CX3CR1**	**Rn02134446_s1**				
**CCL27**	**Rn01437034_m1**		**D6**	**Rn02347939_s1**				
CCL28	*Rn00586715_m1*	*Ct>32*	**DARC**	**Rn03037231_s1**				
**CXCL1**	**Rn00578225_m1**							
CXCL2	*Rn00586403_m1*	*Ct>32*						
CXCL3	*Rn01414231_m1*	*Ct>32*						
**CXCL4**	**Rn01768297_g1**							
CXCL5	*Rn00573587_g1*	*Ct>32*						
CXCL7	*Rn00596603_g1*	*Ct>32*						
**CXCL9**	**Rn00595504_m1**							
**CXCL10**	**Rn01413889_g1**							
**CXCL11**	**Rn00788261_g1**							
**CXCL12**	**Rn00573260_m1**							
**CXCL13**	**Rn01450028_m1**							
**CXCL14**	**Rn01441840_m1**							
**CXCL16**	**Rn01496393_m1**							
CXCL17	*Rn01764053_m1*	*Ct>32*						
**XCL1**	**Rn00592605_m1**							
**CX3CL1**	**Rn00593186_m1**							
