# Supplementary material for: Kinetic mRNA Profiling in a Rat Model of Left-Ventricular Hypertrophy Reveals Early Expression of Chemokines and Their Receptors
Source: PLoS One. 2016 Aug 15;11(8):e0161273. doi: 10.1371/journal.pone.0161273 (PMC4985150; doi:10.1371/journal.pone.0161273)
Supplement: S2 Table — Measurements of the left ventricle area and left ventricle wall thickness in sham-operated and aortic-banded animals from D0 to D14 (n = 6 per group). Analysis was performed with the ImageJ software on slides after hematoxylin-eosin staining. (PDF) [file pone.0161273.s007.pdf]

	D0		D1		D3		D5		D7		D14	
	SHAM	*BANDING*	*SHAM*	*BANDING*	*SHAM*	*BANDING*	*SHAM*	*BANDING*	*SHAM*	*BANDING*	*SHAM*	*BANDING*
LV area (mm^2)	23.6±0.7	21.1±3.1	16.9±1.1	18.3±0.7	21.5±1.3	22.2±1.7	23.2±0.9	24.6±2.1	23.0±1.0	25.8±2.2	34.0±1.0	41.6±2.0
LV wall thickness (mm)	1.3±0.01	1.3±0.09	1.1±0.08	1.2±0.06	1.2±0.02	1.5±0.12	1.3±0.04	1.3±0.05	1.3±0.08	1.4±0.06	1.6±0.05	1.9±0.08
